# Molecular and Phenotypic Characterization of MDR and XDR *Aeromonas* spp. Isolated from Hospital Wastewater

**DOI:** 10.3390/pathogens15101002

**Published:** 2026-09-22

**Authors:** Auria del C. López-Hernández, María de la L. Sevilla-González, Miguel Á. Loyola-Cruz, Gustavo E. Lugo-Zamudio, Emilio M. Durán-Manuel, Graciela Castro-Escarpulli, Karla A. Rodríguez-Encinas, Araceli Rojas-Bernabé, Sandra Olivares-Cruz, María de J. Sánchez-Guzmán, Paulina Carpinteyro-Espin, Dulce M. Razo Blanco-Hernández, Claudia C. Calzada-Mendoza, Ricardo D. Romero-Morelos, Briceida López-Martínez, Miguel Plascencia-Espinosa, Benito Hernández-Castellanos, Julio C. Castañeda-Ortega, Clemente Cruz-Cruz, Juan M. Bello-López

**Affiliations:** 1 Hospital Juárez de México, Mexico City 07760, Mexico; 2 Laboratorio de Conducta Humana, Sección de Estudios de Posgrado e Investigación, Escuela Superior de Medicina, Instituto Politécnico Nacional, Mexico City 11340, Mexico; 3 Laboratorio de Investigación Clínica y Ambiental, Departamento de Microbiología, Escuela Nacional de Ciencias Biológicas, Instituto Politécnico Nacional, Mexico City 11340, Mexico; 4 Laboratorio de Señalización Intracelular, Sección de Estudios de Posgrado e Investigación, Escuela Superior de Medicina, Instituto Politécnico Nacional, Mexico City 11340, Mexico; 5 Centro de Investigación en Biotecnología Aplicada del Instituto Politécnico Nacional (CIBA-IPN), Tlaxcala 90700, Mexico; 6 Facultad de Biología, Universidad Veracruzana, Xalapa 91090, Mexico

**Keywords:** antimicrobial resistance, *Aeromonas*, wastewater, carbapenems, cephalosporins

## Abstract

Hospital wastewater (HWW) is increasingly recognized as an environmental hotspot for the selection and dissemination of antimicrobial resistance (AMR). Although surveillance programs primarily target ESKAPE pathogens, the contribution of environmental bacteria such as *Aeromonas* spp. remains poorly characterized. The aim of this work was to investigate antimicrobial resistance mechanisms, focusing on carbapenem and cephalosporin resistance, in *Aeromonas* spp. recovered from HWW. Thirty *Aeromonas* isolates were identified by *rpoD* restriction fragment length polymorphism analysis and characterized by antimicrobial susceptibility testing, detection of β-lactam resistance genes, class 1 integrons, and genetic relatedness using ERIC-PCR fingerprinting. All isolates exhibited operationally defined multidrug-resistant (MDR) or extensively drug-resistant (XDR) phenotypes, with resistance predominating against cephalosporins, carbapenems, and trimethoprim–sulfamethoxazole. The clinically relevant gene families/groups *bla_KPC_* family, *bla_OXA-_*_48_-like, *bla_CTX-M_* group 1, and *bla_SHV_* were detected by PCR, whereas *bla_NDM_*, *bla_VIM_*, *bla_IMP_*, and *mcr-1* were absent. Class 1 integrons were identified in 36.7% of isolates, revealing diverse cassette arrays carrying *dfrA* and *aadA* resistance genes. ERIC-PCR demonstrated high fingerprint diversity with limited genetic relatedness. Microbiological surveillance of HWW revealed that *Aeromonas* populations constitute an environmental reservoir of clinically relevant β-lactam resistance determinants, supporting further evaluation of their potential role in AMR surveillance strategies at the hospital–environment interface.

## 1. Introduction

Antimicrobial resistance (AMR) has emerged as one of the greatest threats to global public health because it compromises the effectiveness of last-line antibiotics and severely limits therapeutic options for the treatment of bacterial infections [1]. Traditionally, the emergence and spread of AMR have been attributed to the inappropriate and excessive use of antimicrobials in both hospital and community settings. However, increasing evidence indicates that additional environmental reservoirs also play a substantial role in the maintenance and dissemination of resistance determinants. Among these, hospital wastewater (HWW) has attracted considerable attention because it continuously receives antimicrobial residues together with resistant microorganisms, including members of the ESKAPE group (*Enterococcus faecium*, *Staphylococcus aureus*, *Klebsiella pneumoniae*, *Acinetobacter baumannii*, *Pseudomonas aeruginosa*, and *Enterobacter* spp.) [2,3,4,5].

Consequently, HWW is recognized as a complex ecosystem where pathogenic bacteria, antimicrobial compounds, heavy metals, and organic matter coexist, creating favorable conditions for the selection of resistant bacteria and horizontal gene transfer [3,6,7,8]. For this reason, microbiological surveillance studies in HWW have predominantly focused on ESKAPE pathogens because of their well-established role in healthcare-associated and community-acquired infections [9,10,11,12,13,14,15,16]. Nevertheless, recent evidence suggests that environmental aquatic bacteria also actively participate in the environmental dissemination of AMR, particularly resistance to β-lactam antibiotics such as carbapenems and cephalosporins, which represent critical therapeutic options for the treatment of infections caused by multidrug-resistant bacteria [17,18,19,20]. Among these environmental bacteria, the genus *Aeromonas* has gained increasing attention because of its ubiquitous distribution in aquatic environments, persistence in drainage systems and water treatment processes, and its ability to cause both community-acquired and healthcare-associated infections [21,22,23,24,25]. In addition, several studies have demonstrated that *Aeromonas* spp. isolated from environments impacted by human activities frequently harbor antimicrobial resistance determinants commonly associated with clinically relevant bacterial pathogens [26,27,28].

In a previous study, we demonstrated that *Aeromonas* spp. coexist with antimicrobial-resistant ESKAPE pathogens in HWW containing residues of clinically relevant antibiotics, including meropenem, ceftazidime, and cefepime [3]. That study was based on a 12-month monitoring program conducted at five HWW discharge points and provided a broad assessment of the microbiological risks associated with the release of antibiotic-resistant bacteria together with β-lactam antibiotic residues. *Aeromonas* spp. were recovered as part of the resistant Gram-negative bacterial collection generated during that surveillance. This ecological coexistence suggests that HWW may facilitate horizontal gene transfer between environmental *Aeromonas* populations and clinically important bacteria. Supporting this hypothesis, carbapenemase-producing *Aeromonas* carrying *bla_KPC_* have previously been recovered from hospital wastewater together with ESKAPE pathogens harboring the same resistance determinant [29]. Likewise, environmental *Aeromonas* isolates carrying *bla_KPC-_*_2_ have been reported in urban wastewater systems, indicating that these clinically relevant resistance genes can persist and disseminate beyond hospital settings [30]. Because of its ecological adaptability, environmental persistence, and remarkable capacity to acquire, maintain, and disseminate antimicrobial resistance determinants, *Aeromonas* has recently been proposed as a sentinel microorganism for AMR surveillance [31]. These characteristics place this genus at the interface between environmental and clinical reservoirs of antimicrobial resistance.

Therefore, investigating antimicrobial resistance in *Aeromonas* isolated from HWW may provide valuable insights into its contribution to the environmental maintenance and dissemination of clinically relevant resistance determinants. Accordingly, rather than proposing a new conceptual framework for environmental AMR dissemination, this study provides a regionally specific characterization of antimicrobial resistance in *Aeromonas* populations recovered from HWW in Mexico City, integrating species assignment, resistance phenotypes, clinically relevant β-lactam resistance determinants, class 1 integron structures, and ERIC-PCR fingerprinting. The aim of this study was to characterize antimicrobial resistance mechanisms, with particular emphasis on β-lactam resistance (carbapenems and cephalosporins), in *Aeromonas* spp. isolated from HWW and to discuss their potential role as environmental reservoirs of antimicrobial resistance genes at the hospital–environment interface.

## 2. Materials and Methods

### 2.1. Origin of Aeromonas spp. Strains and Identification by rpoD-RFLP

A total of 30 *Aeromonas* spp. isolates recovered from HWW at Hospital Juárez de México (HJM) were included in this study. The isolates belonged to a previously described strain collection [3]. All *Aeromonas* isolates available in the original resistant bacterial collection were included; no additional selection based on species, resistance phenotype, sampling month, or discharge point was applied. Briefly, the original wastewater monitoring was conducted monthly from January to December 2024 at the five hospital wastewater discharge points, yielding a total of 60 wastewater samples. Samples were collected at a standardized morning time (10:00 a.m.), transported at 4 °C, and processed as previously described [3]. Resistant Gram-negative bacteria were recovered on MacConkey agar supplemented with the corresponding selective antimicrobial agents; representative resistant colonies were subsequently purified and identified by MALDI-TOF MS. The 30 *Aeromonas* isolates analyzed here originated from this previously established resistant Gram-negative bacterial collection.

Initial species identification was performed by matrix-assisted laser desorption/ionization time-of-flight mass spectrometry (MALDI-TOF MS). However, because MALDI-TOF databases have limited discriminatory power for discriminating closely related *Aeromonas* species, particularly within taxonomically complex groups, species identification was further assessed by restriction fragment length polymorphism (RFLP) analysis of the housekeeping *rpoD* gene following the method described by Puah et al. (2018) [32]. Briefly, genomic DNA was extracted from each isolate, and a conserved *rpoD* fragment was amplified by PCR using degenerate primers (Table 1). The amplicons (approximately 770–782 bp) were verified by agarose gel electrophoresis and digested with the restriction enzyme AluI. Restriction profiles were analyzed by agarose gel electrophoresis, and species assignments were established according to the species-specific restriction patterns described by Puah et al. [32].

### 2.2. Antimicrobial Resistance Profiles

Antimicrobial susceptibility of *Aeromonas* spp. isolates was determined according to the Clinical and Laboratory Standards Institute (CLSI) M45 guidelines for infrequently isolated or fastidious bacteria [38]. Susceptibility testing was performed by the disk diffusion method on Mueller–Hinton agar using the interpretive criteria recommended for *Aeromonas* spp. The following antimicrobial agents were evaluated: imipenem (10 µg), meropenem (10 µg), ertapenem (10 µg), cefoxitin (30 µg), ceftazidime (30 µg), ceftriaxone (30 µg), cefotaxime (30 µg), cefepime (30 µg), gentamicin (10 µg), amikacin (30 µg), ciprofloxacin (5 µg), trimethoprim–sulfamethoxazole (1.25/23.75 µg), and colistin. The CLSI M45 susceptible/intermediate/resistant breakpoints were applied to the disk-diffusion results for carbapenems, cephalosporins, aminoglycosides, ciprofloxacin, and trimethoprim–sulfamethoxazole [38], and the agent-specific zone-diameter thresholds were verified against the corresponding CLSI criteria. Because disk diffusion is not recommended for colistin, susceptibility to this antimicrobial was determined by broth microdilution following CLSI M100 guidelines [39]. The absence of *Aeromonas*-specific CLSI interpretive criteria for colistin was considered when interpreting these results. *Escherichia coli* ATCC 25922 and *Pseudomonas aeruginosa* ATCC 27853 were included as quality control strains. These reference strains were used exclusively for quality control of antimicrobial susceptibility testing and were not used as biological comparators to define resistance in *Aeromonas* spp.

Antimicrobial resistance profiles were operationally classified as multidrug-resistant (MDR), extensively drug-resistant (XDR), or pandrug-resistant (PDR) according to the international consensus definitions proposed by Magiorakos et al. (2012) [40]. For this classification, the antimicrobial agents evaluated were organized into six categories: carbapenems, cephalosporins, aminoglycosides, fluoroquinolones, folate pathway inhibitors, and polymyxins. For the operational MDR/XDR/PDR classification only, isolates with intermediate susceptibility were grouped as non-susceptible, as recommended in the original consensus document. Isolates were classified as MDR when they showed non-susceptibility to one or more antimicrobial agents across at least three different antimicrobial categories. XDR was assigned when non-susceptibility extended to nearly all evaluated categories, with activity retained in no more than two categories. Isolates exhibiting non-susceptibility to every antimicrobial agent assessed across all tested categories were categorized as PDR. Because these consensus definitions were not originally developed specifically for *Aeromonas* spp., MDR and XDR were used here as operational phenotypic descriptors to standardize resistance-pattern comparisons. Intrinsic resistance in *Aeromonas* is species- and antimicrobial-dependent and may involve chromosomally encoded β-lactamases; therefore, phenotypic resistance alone was not interpreted as evidence of an acquired resistance determinant.

### 2.3. Carbapenemases and ESBL Production and Their Relationship with Genotypes

Carbapenem-resistant *Aeromonas* isolates were screened for phenotypic carbapenemase activity using the modified carbapenem inactivation method (mCIM) according to Pierce et al. (2017) [41]. Because mCIM detects carbapenem hydrolysis but does not identify the specific enzyme responsible, the assay was not interpreted as distinguishing intrinsic CphA activity from acquired carbapenemase mechanisms. ESBL production was evaluated by the combination disk method using ceftazidime (30 μg), cefotaxime (30 μg), ceftazidime/clavulanic acid (30/10 μg), and cefotaxime/clavulanic acid (30/10 μg), following CLSI recommendations. *Klebsiella pneumoniae* carrying *bla_NDM-1_* and *K. pneumoniae* ATCC 700603 were used as positive controls for carbapenemase and ESBL production, respectively, whereas *E. coli* ATCC 25922 served as the negative control.

### 2.4. Detection of Carbapenemase, ESBL, and Colistin Resistance Genes

Carbapenemase- and ESBL-producing *Aeromonas* isolates were screened by end-point PCR for the detection of metallo-β-lactamase genes (*bla_NDM_*, *bla_VIM_*, and *bla_IMP_*), serine carbapenemase gene families/groups (*bla*_KPC_ and *bla*_OXA-48_-like) and ESBL genes (*bla_SHV_* and *bla_CTX-M_* group 1) using the primers listed in Table 1. PCR amplification conditions for each target were performed according to the protocols described in the corresponding original references, without modification. In addition, the plasmid-mediated colistin resistance gene (*mcr-1*) was investigated according to the protocol described by Rebelo et al. (2018) [35]. PCR products were separated by horizontal electrophoresis on 2.0% agarose gels prepared with 1× TBE buffer, visualized under UV illumination, and compared with an appropriate molecular weight marker. Previously characterized gene-positive strains were included as positive controls in the corresponding PCR assays. Positive controls for the carbapenemase gene targets were obtained from the previously characterized strains described by Cortés-Ortíz et al. (2021) [42] and Loyola-Cruz et al. (2023) [43]. *Klebsiella pneumoniae* ATCC 700603 and *Escherichia coli* BAA-3287 were used as positive controls for *bla*_SHV_ and *bla*_CTX-M_ group 1, respectively. Positive-control amplification was verified in the corresponding PCR runs before interpretation of the *Aeromonas* isolates. Because the β-lactamase amplicons were not sequenced, PCR-positive results were interpreted at the gene-family/group level rather than as sequence-confirmed allelic variants.

### 2.5. Detection of Class 1 Integrons and Their Gene Cassettes

All *Aeromonas* isolates were examined for genetic elements associated with class 1 integrons. PCR assays targeted the 5′ integrase gene (*intI1*), the cassette-containing variable region, and the 3′ conserved segment comprising *qacEΔ1-sul1*, using the primer sets listed in Table 1. The amplified variable regions were subsequently sequenced at the Institute of Biology, UNAM, on a 3730xl DNA Analyzer (Applied Biosystems, Foster City, CA, USA) using the in-F and in-B primers. Resistance gene cassettes were identified by comparing the resulting nucleotide sequences against GenBank entries through the BLASTX algorithm (http://blast.ncbi.nlm.nih.gov (accessed on 10 July 2026)). *Aeromonas salmonicida* 718 harboring plasmid pRAS1 [*IncU*; class 1 integron (*intI1-dfrA16-qacEΔ1/sul1*)] served as the positive control [44].

### 2.6. Molecular Typing of Aeromonas spp. Strains by ERIC-PCR

*Aeromonas* strains were analyzed by enterobacterial repetitive intergenic consensus (ERIC)-PCR to evaluate genetic relatedness based on fingerprinting profiles [37]. Banding-pattern comparison was based on the presence or absence of amplified intergenic fragments. Bands were assigned according to their electrophoretic position and apparent molecular size within each gel, and each band position was coded as present (1) or absent (0) for each isolate to generate a binary data matrix. Genetic profiles were analyzed by intra-gel pattern comparison using ImageLab 5.2.1. To confirm the reproducibility of assays, they were performed three times. The Tenover criteria [45] were used as an interpretative framework for comparing ERIC-PCR banding patterns rather than as definitive evidence of clonality. No predefined percentage similarity threshold was used to define clonality; ERIC-PCR profiles were interpreted qualitatively using the Tenover framework based on band-pattern differences. Isolates showing indistinguishable ERIC-PCR profiles were considered closely related, whereas isolates with different banding patterns were considered genetically distinct. Genetic relatedness among the isolates was represented using a distance matrix generated with a linear semilogarithmic approach. Cluster analysis was performed by the unweighted pair group method with arithmetic mean (UPGMA), applying the Dice coefficient to estimate similarity. The stability of the resulting clusters was assessed by bootstrap analysis with 1000 replicates in PAST software, version 4.09.

## 3. Results

### 3.1. Species Identification of Aeromonas Isolates by rpoD-RFLP Analysis

Species assignment of the 30 *Aeromonas* isolates recovered from HWW was performed by *rpoD*-RFLP analysis. Electrophoretic restriction patterns identified six *Aeromonas* species, with *A. caviae* being the most frequently represented species (*n* = 14), followed by *A. media* (*n* = 5), *A. veronii* (*n* = 4), *A. salmonicida* (*n* = 4), *A. dhakensis* (*n* = 2), and *A. jandaei* (*n* = 1). *rpoD*-RFLP analysis revealed discrepancies with the initial MALDI-TOF MS identification. Specifically, one isolate initially identified as *A. hydrophila* was reclassified as *A. media*, whereas two isolates previously identified as *A. caviae* were reassigned to *A. dhakensis*.

### 3.2. Antimicrobial Susceptibility Profiles of Aeromonas Isolates from HWW

Operational MDR and XDR phenotypes were identified among the *Aeromonas* isolates, whereas no PDR isolates were detected. Overall, 11/30 (36.7%) isolates were classified as MDR and 19/30 (63.3%) as XDR. XDR phenotypes predominated in *A. caviae* (9/14, 64.3%), followed by *A. media* (4/5, 80.0%), *A. veronii* (2/4, 50.0%), *A. salmonicida* (1/4, 25.0%), *A. dhakensis* (2/2, 100%), and *A. jandaei* (1/1, 100%). MDR phenotypes were identified in *A. caviae* (5/14, 35.7%), *A. media* (1/5, 20.0%), *A. veronii* (2/4, 50.0%) and *A. salmonicida* (3/4, 75.0%). Analysis of antimicrobial susceptibility by antimicrobial class revealed that resistance was most frequently observed against β-lactams (cephalosporins and carbapenems) and folate pathway inhibitors, whereas lower resistance rates were detected for aminoglycosides, ciprofloxacin, and colistin (Figure 1).

Among the cephalosporins, cefotaxime and ceftriaxone exhibited the highest resistance frequencies, particularly in *A. caviae* and *A. media*, the two most frequently represented species in this collection. All isolates of *A. caviae*, *A. media*, *A. veronii*, *A. dhakensis*, and *A. jandaei* exhibited resistance to at least one cephalosporin, whereas among *A. salmonicida*, only one isolate (25%) exhibited cephalosporin resistance, and this isolate was resistant to all five cephalosporins evaluated. Notably, *A. media* exhibited consistently high resistance rates to all cephalosporins tested. Intermediate susceptibility to cefepime and cefoxitin was observed in *A. veronii* and *A. salmonicida*, with the latter species showing the highest susceptibility to these antimicrobials (Figure 1). Resistance to carbapenems was most frequently observed for ertapenem (46.7%, 14/30) and meropenem (40.0%, 12/30).

Overall, 20 isolates (66.7%, 20/30) were resistant to at least one carbapenem, predominantly among *A. caviae* and *A. media*. In contrast, imipenem showed the lowest carbapenem resistance frequency (20.0%, 6/30), with 40.0% (12/30) of isolates showing intermediate susceptibility and 40.0% (12/30) remaining susceptible. Only one *A. veronii* isolate (AV-199) exhibited resistance to all three carbapenems tested. Among aminoglycosides, resistance to amikacin (26.7%, 8/30) was more frequent than resistance to gentamicin (3.3%, 1/30), although intermediate susceptibility to these agents was also observed. Ciprofloxacin, the only fluoroquinolone evaluated, showed resistance in 46.7% (14/30) of the isolates, intermediate susceptibility in 36.7% (11/30), and susceptibility in 16.7% (5/30). Trimethoprim–sulfamethoxazole exhibited resistance in 80.0% (24/30) of the isolates, representing one of the highest resistance frequencies observed among the antimicrobial agents tested. Notably, all *A. media* isolates were resistant to this antimicrobial. Colistin resistance was detected in 16.7% (5/30) of the isolates, specifically *A. caviae* AC-048, *A. media* AM-249, and *A. salmonicida* AS-035, AS-008, and AS-016, whereas 63.3% (19/30) remained susceptible (Figure 1).

### 3.3. Distribution of Antimicrobial Resistance Genes Among Aeromonas Isolates

Genotypic characterization of resistant isolates revealed a heterogeneous distribution of β-lactam resistance genes (Figure 1). Among carbapenemase genes, the serine β-lactamases *bla_KPC_* family (20%) and *bla_OXA-_*_48_-like (13.3%) were detected by PCR. Among the ESBL genes investigated, *bla_CTX-M_* group 1 was the most frequently identified (30%), followed by *bla_SHV_* (6.6%). Analysis by species showed that *A. caviae* and *A. media*, the two most frequently represented species in this collection, accounted for most *blaKPC* family, *bla_CTX-M_* group 1, and *bla_SHV_*-positive isolates. Notably, one *A. caviae* isolate (AC-114) simultaneously carried all three resistance gene families/group. In the remaining species, β-lactam resistance genes were detected less frequently, although *bla_OXA-_*_48_-like was identified in three *A. salmonicida* isolates and one *A. jandaei* isolate. Two additional gene combinations were observed: the coexistence of *bla_CTX-M_* group 1 and *bla_SHV_* in *A. dhakensis* (AD-089), and the coexistence of *bla_OXA-_*_48_-like and *bla_CTX-M_* group 1 in *A. jandaei* (AJ-004). None of the isolates carried *bla_IMP_*, *bla_VIM_*, *bla_NDM_*, or the plasmid-mediated colistin resistance gene *mcr-1* (Figure 1).

### 3.4. Structural Organization and Gene Cassette Arrays of Class 1 Integrons

Class 1 integrons were detected in 11 of the 30 *Aeromonas* isolates analyzed (36.7%). The distribution of these mobile genetic elements varied among species, with the highest frequency observed in *A. caviae* (*n* = 5), followed by *A. media* (*n* = 3), *A. salmonicida* (*n* = 2), and *A. jandaei* (*n* = 1), whereas no class 1 integrons were detected in *A. veronii* or *A. dhakensis*. Structural analysis identified two integron configurations. Six isolates carried cassette-containing class 1 integrons comprising the *intI1–variable region–qacEΔ1–sul1* structure, whereas five isolates harbored class 1 integrons lacking gene cassettes, characterized by the presence of the conserved 5′ and 3′ segments separated by a variable region of approximately 150 bp. Cassette-containing integrons were identified in *A. caviae*, *A. salmonicida*, and *A. jandaei*, whereas cassette-free integrons predominated in *A. media*. Notably, cassette-containing integrons were predominantly detected among isolates exhibiting an XDR phenotype. Sequence analysis of the variable region identified five distinct gene cassette arrays and one cassette-free class 1 integron configuration (Figure 2).

These arrays contained resistance genes conferring resistance to trimethoprim and aminoglycosides. Trimethoprim resistance was associated with members of the *dfrA* gene family encoding dihydrofolate reductases, including the *dfrA5*, *dfrA12*, *dfrA17*, *dfrA48*, and *dfrA7* variants. Aminoglycoside resistance genes belonged to the *aadA* family encoding aminoglycoside adenylyltransferases and comprised the *aadA1*, *aadA2*, and *aadA5* variants. Three gene cassette arrays containing two resistance genes (*dfrA17-aadA5*, *dfrA12-aadA2*, and *dfrA7-aadA1*) and two arrays containing a single resistance gene (*dfrA5* or *dfrA48*) were identified (Figure 2). In addition, a sixth structural configuration lacked gene cassettes and contained only the conserved regions of the class 1 integron. Representative nucleotide sequences for each of the five distinct cassette-containing class 1 integron arrangements were deposited in GenBank under accession numbers PZ914413, PZ914414, PZ914415, PZ914416, and PZ914417. Collectively, these findings reveal considerable structural diversity among class 1 integrons and their associated gene cassette arrays in the *Aeromonas* isolates analyzed.

### 3.5. ERIC-PCR Fingerprint Diversity and Genetic Relatedness of Aeromonas spp. from HWW

ERIC-PCR analysis of the 29 *Aeromonas* isolates recovered from HWW revealed variable levels of fingerprint diversity and genetic relatedness among the species analyzed. The dendrograms shown in Figure 3 identified groups of closely related isolates in four of the five species. *Aeromonas jandaei* was excluded from the analysis because only a single isolate was available. *Aeromonas caviae* exhibited the greatest ERIC-PCR fingerprint diversity, with highly heterogeneous ERIC-PCR fingerprinting patterns. Although isolates AC-127 and AC-231 clustered together, forming a single closely related group, the remaining isolates displayed distinct fingerprinting profiles, indicating marked genetic heterogeneity based on ERIC-PCR patterns. In *A. media*, isolates AM-049, AM-052, and AM-053 showed indistinguishable ERIC-PCR profiles with 100% similarity, whereas AM-246 and AM-249 showed closely related ERIC-PCR profiles but remained outside the closely related cluster. Likewise, isolates AV-223 and AV-258 of *A. veronii* grouped together, exhibiting high genetic similarity and forming a closely related cluster.

Among the *A. salmonicida* isolates, AS-006, AS-008, and AS-016 clustered together with high genetic similarity, whereas isolate AS-035 exhibited a distinct fingerprinting profile. In contrast, the two *A. dhakensis* isolates (AD-089 and AD-180) displayed different ERIC-PCR profiles and did not form a closely related cluster.

## 4. Discussion

In recent years, HWW has been recognized as an important ecological niche for the selection and dissemination of AMR because it receives a complex mixture of hospital-associated microorganisms, environmental bacteria, antimicrobial residues, and other chemical contaminants [46,47]. Consequently, most bacteriological surveillance studies conducted in HWW have focused on ESKAPE pathogens because of their clinical relevance and environmental impact [3,10]. In contrast, considerably less attention has been paid to the influence of hospital-derived microorganisms on indigenous aquatic bacteria such as members of the genus *Aeromonas*. The coexistence of these bacterial populations within the same ecological niche creates favorable conditions for horizontal gene transfer, potentially enabling *Aeromonas* to act as both a reservoir and a vehicle for the dissemination of AMR determinants. Discrepancies were observed between the initial MALDI-TOF MS identification and the subsequent *rpoD*-RFLP species assignments, including the reassignment of isolates initially identified as *A. hydrophila* or *A. caviae*. These findings highlight the limitations of MALDI-TOF MS for species-level discrimination within the genus *Aeromonas*, particularly among closely related taxa. Although MALDI-TOF MS remains a useful approach for routine bacterial identification, complementary molecular methods may be necessary when accurate species-level identification of *Aeromonas* is required. Although *rpoD*-RFLP provides species-level discrimination according to the validated method used [32], sequencing of *rpoD* or higher-resolution molecular approaches would provide additional taxonomic confirmation, particularly for closely related *Aeromonas* species.

The present study focused on HWW; however, antimicrobial resistance in *Aeromonas* should not be considered a phenomenon restricted to hospital effluents. *Aeromonas* spp. are widely distributed in aquatic environments and have also been reported in urban wastewater treatment systems, freshwater-associated environments, and agricultural settings [22,25,30]. Thus, HWW should be viewed as one relevant component of a broader environmental context in which *Aeromonas* may harbor antimicrobial resistance determinants. Comparative studies involving hospital, municipal, agricultural, and natural aquatic environments are needed to better define the relative contribution of these settings to environmental AMR.

The findings of the present study suggest that several *Aeromonas* species may contribute to the acquisition and persistence of hospital-associated AMR in HWW. All isolates exhibited either operational MDR or XDR phenotypes (Figure 1), and several carried genes encoding carbapenemases and ESBLs, resistance determinants that have historically been associated with clinically important nosocomial pathogens [18,19]. Although antimicrobial resistance has previously been reported in environmental *Aeromonas* isolates [31,48], the high proportion of MDR and XDR phenotypes observed here should be interpreted within the context of the study design, because the analyzed isolates originated from a previously established collection of antimicrobial-resistant Gram-negative bacteria. Therefore, these frequencies describe the analyzed *Aeromonas* collection and should not be interpreted as prevalence estimates for the overall *Aeromonas* population in HWW. In addition, the relatively small number of isolates and the uneven representation of species, including species represented by only one or two isolates, limit species-level comparisons and the generalizability of the findings. Therefore, the potential incorporation of *Aeromonas* into routine AMR surveillance should be considered a hypothesis for further evaluation in larger and more representative studies rather than a recommendation derived solely from the present dataset. The predominance of XDR phenotypes among *A. caviae* isolates is particularly noteworthy because this species is one of the *Aeromonas* species most frequently associated with human infections and antimicrobial resistance [23,49].

Likewise, the results obtained for *A. media* deserve attention. Although this species has received comparatively little attention, several isolates displayed resistance to cephalosporins, a high frequency of XDR phenotypes, and the presence of class 1 integrons. Information regarding the contribution of *A. media* to the dissemination of AMR, particularly in HWW, remains scarce, making direct comparisons with previous reports difficult. Nevertheless, our findings suggest that this species may also constitute an environmental reservoir of antimicrobial resistance determinants [19]. The less frequently isolated species, including *A. veronii*, *A. salmonicida*, *A. dhakensis*, and *A. jandaei*, also exhibited MDR and XDR phenotypes, primarily associated with resistance to carbapenems and cephalosporins. Although susceptible *A. veronii* strains have been reported as causative agents of cellulitis, bacteremia, and sepsis [50], the identification of MDR and XDR isolates raises additional clinical concerns, as infections caused by resistant strains could compromise therapeutic efficacy, particularly in immunocompromised patients. Traditionally regarded as a fish pathogen, *A. salmonicida* has increasingly been recognized as an opportunistic human pathogen responsible for bacteremia, urinary tract infections, and peritonitis, predominantly in immunocompromised individuals [51].

In the present study, three of the four *A. salmonicida* isolates exhibited an MDR phenotype, whereas the remaining isolate was classified as XDR, with resistance to both carbapenems and cephalosporins. These findings are clinically relevant because most reported human infections caused by *A. salmonicida* have been successfully treated with these antimicrobial agents [52,53]. Therefore, the emergence of MDR and XDR strains may substantially reduce therapeutic options for similar infections. Furthermore, the recovery of resistant *A. salmonicida* isolates from HWW supports the hypothesis that this species may serve as an environmental reservoir of clinically relevant antimicrobial resistance determinants with potential public health implications [54]. Finally, both *A. dhakensis* and *A. jandaei* exclusively exhibited XDR phenotypes. *Aeromonas dhakensis* is considered one of the most virulent species within the genus and has frequently been associated with bacteremia, necrotizing fasciitis, and other invasive infections [55]. Although *A. jandaei* has been reported less frequently in human infections, the XDR phenotype identified in the present study suggests that this species may also represent an environmental reservoir of clinically relevant antimicrobial resistance determinants [56].

Resistance to cephalosporins was one of the most relevant findings of the present study, as all *Aeromonas* species analyzed exhibited resistance to at least one cephalosporin, with cefotaxime and ceftriaxone being the most frequently affected agents (Figure 1). This phenotypic profile was consistent with the detection of the *bla_CTX-M_* group 1 and *bla_SHV_* genes, two of the most widespread genetic determinants among ESBL-producing bacteria [57,58]. The occurrence of these genes in environmental *Aeromonas* species has been reported previously, although at considerably lower frequencies than those described for clinically relevant members of the *Enterobacterales* [57,59]. Carbapenem resistance deserves particular attention because these antimicrobials are considered last-resort therapeutic agents for the treatment of infections caused by multidrug-resistant bacteria. As shown in Figure 1, more than 60% of the isolates were resistant to at least one of the carbapenems tested, and the *bla_KPC_* family and *bla_OXA-_*_48_-like genes were detected in several *Aeromonas* species by PCR. Because these β-lactamase amplicons were not sequence-confirmed, these findings should be interpreted as PCR-based detection at the gene-family/group level; sequencing would be required to establish specific allelic variants. Although intrinsic carbapenem resistance in *Aeromonas* has traditionally been attributed to chromosomally encoded metallo-β-lactamases such as CphA [49], mCIM detects phenotypic carbapenemase activity [36] but cannot by itself distinguish intrinsic CphA-mediated activity from that produced by acquired carbapenemases. Therefore, mCIM results were interpreted together with the PCR detection of the carbapenemase gene families/groups investigated in this study. The contribution of CphA was not directly evaluated molecularly and should therefore be considered a limitation when interpreting the phenotypic carbapenemase results. The detection of these carbapenemase gene families/groups is of greater epidemiological concern. These determinants have historically been associated with *Enterobacterales* and currently represent one of the principal mechanisms responsible for carbapenem resistance in hospital settings worldwide [60].

The detection of *bla_KPC_*-family-positive *Aeromonas* isolates in HWW is not an isolated observation. Picão et al. (2013) reported KPC-producing *Aeromonas* isolates recovered from Brazilian hospital effluents, demonstrating that aquatic bacteria may participate in the environmental circulation of resistance genes traditionally associated with clinical pathogens [29]. Likewise, Sekizuka et al. (2019) identified environmental carbapenemase-producing bacteria in urban aquatic systems, providing further evidence that these resistance determinants can persist outside hospital environments [30]. Although horizontal gene transfer was not evaluated in the present study, the detection of resistance genes typically associated with hospital pathogens in environmental *Aeromonas* isolates reinforces the hypothesis that genetic exchange may occur within HWW ecosystems. The mobilome of *Aeromonas* has been recognized as an important driver of antimicrobial resistance acquisition and dissemination. Previous studies have demonstrated that conjugative plasmids, transposons, genomic islands, and integrons contribute to the capture and horizontal transfer of resistance determinants within this genus [61].

Accordingly, *Aeromonas* may participate in horizontal gene-transfer networks in wastewater environments; however, the present study does not establish this genus as a key driver of horizontal gene transfer. Class 1 integrons are also recognized as mobile genetic platforms associated with antimicrobial resistance determinants in Gram-negative bacteria [62,63]. However, the genetic context of the *bla_KPC_* family and *bla_OXA-_*_48_-like genes detected in the present study was not determined. Because plasmid profiling, conjugation or transformation assays, and whole-genome sequencing were not performed, their chromosomal or plasmid location and their potential mobility cannot be established from the present data. Thus, any association of these genes with mobile genetic elements remains hypothetical and represents a limitation of the study.

Class 1 integrons were detected in 36.7% of the isolates analyzed. However, none of the gene cassette arrays identified in the variable region contained carbapenemase- or ESBL-associated genes. Instead, all cassette arrays encoded resistance to trimethoprim and aminoglycosides through variants of the *dfrA* and *aadA* gene families. Similar findings have previously been reported by our research group in *Aeromonas*, in which class 1 integrons carried gene cassettes encoding resistance to aminoglycosides and folate pathway inhibitors [21,64]. These observations indicate that the class 1 integrons characterized in this study did not carry the β-lactam resistance determinants detected by PCR. However, the genetic location of these β-lactamase genes could not be established using the methods employed in the present study. An additional finding was the detection of class 1 integrons lacking gene cassettes. These structures retain the conserved recombination platform and therefore remain competent to acquire resistance cassettes available in the surrounding environment [64].

Gillings (2014) proposed that such integrons represent evolutionary platforms capable of capturing new resistance determinants under appropriate selective conditions [65]. Considering the continuous exposure of HWW to cephalosporins, carbapenems, and other antimicrobial agents, these cassette-free integrons may constitute genetic platforms for the future acquisition of clinically relevant resistance genes. Taken together, our findings demonstrate the presence of MDR and XDR *Aeromonas* isolates harboring clinically relevant β-lactam resistance determinants and class 1 integrons in HWW. These findings support the hypothesis that *Aeromonas* may serve as an environmental reservoir of antimicrobial resistance at the hospital–environment interface; however, the extent and mechanisms of resistance-gene dissemination require further investigation using approaches such as conjugation assays, plasmid characterization, and whole-genome sequencing.

## 5. Conclusions

The occurrence of operationally defined MDR and XDR phenotypes, together with the detection of carbapenemase- and ESBL-associated genes, class 1 integrons, and ERIC-PCR fingerprint diversity, supports the potential role of *Aeromonas* populations inhabiting HWW as environmental reservoirs of clinically relevant antimicrobial resistance determinants. The coexistence of resistant *Aeromonas* spp. and ESKAPE pathogens within HWW supports further evaluation of environmental bacteria as potential components of antimicrobial resistance surveillance strategies. However, the present findings do not demonstrate active horizontal or plasmid-mediated dissemination or establish direct links with clinical transmission; these aspects require further investigation using conjugation assays and whole-genome sequencing. Such an approach may improve our understanding of the environmental dissemination of antimicrobial resistance at the hospital–environment interface.

## Figures and Tables

**Figure 1:**
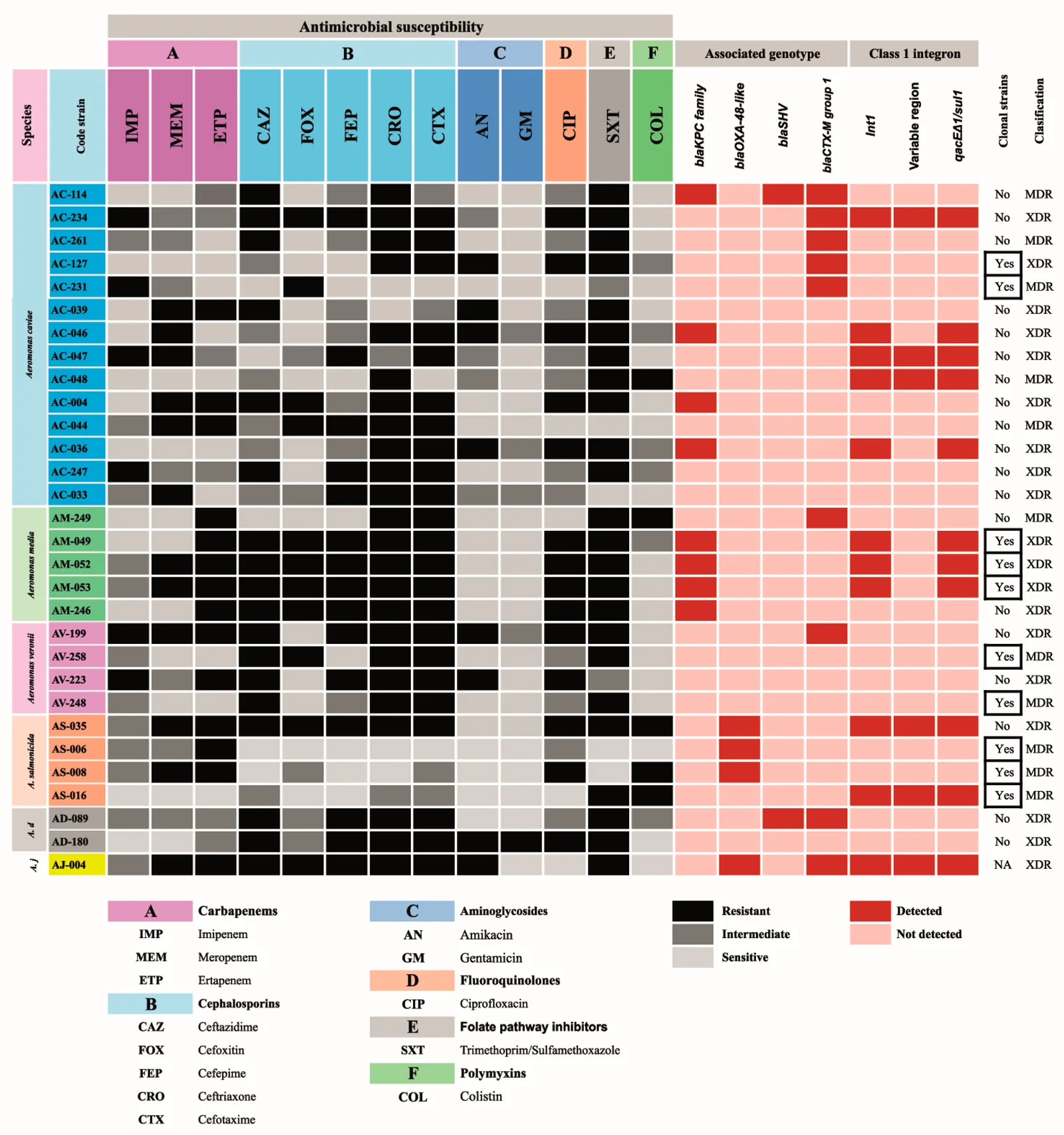
Antimicrobial susceptibility heatmap showing resistance phenotypes, resistance-associated genes, class 1 integrons, and MDR/XDR classification of *Aeromonas* spp. strains isolated from HWW. MDR and XDR phenotypes were operationally defined using the criteria proposed by Magiorakos et al. (2012) [40], as described in Section 2.2. Susceptibility is displayed as S, I, and R; intermediate results were considered non-susceptible only for the operational MDR/XDR/PDR classification.

**Figure 2:**
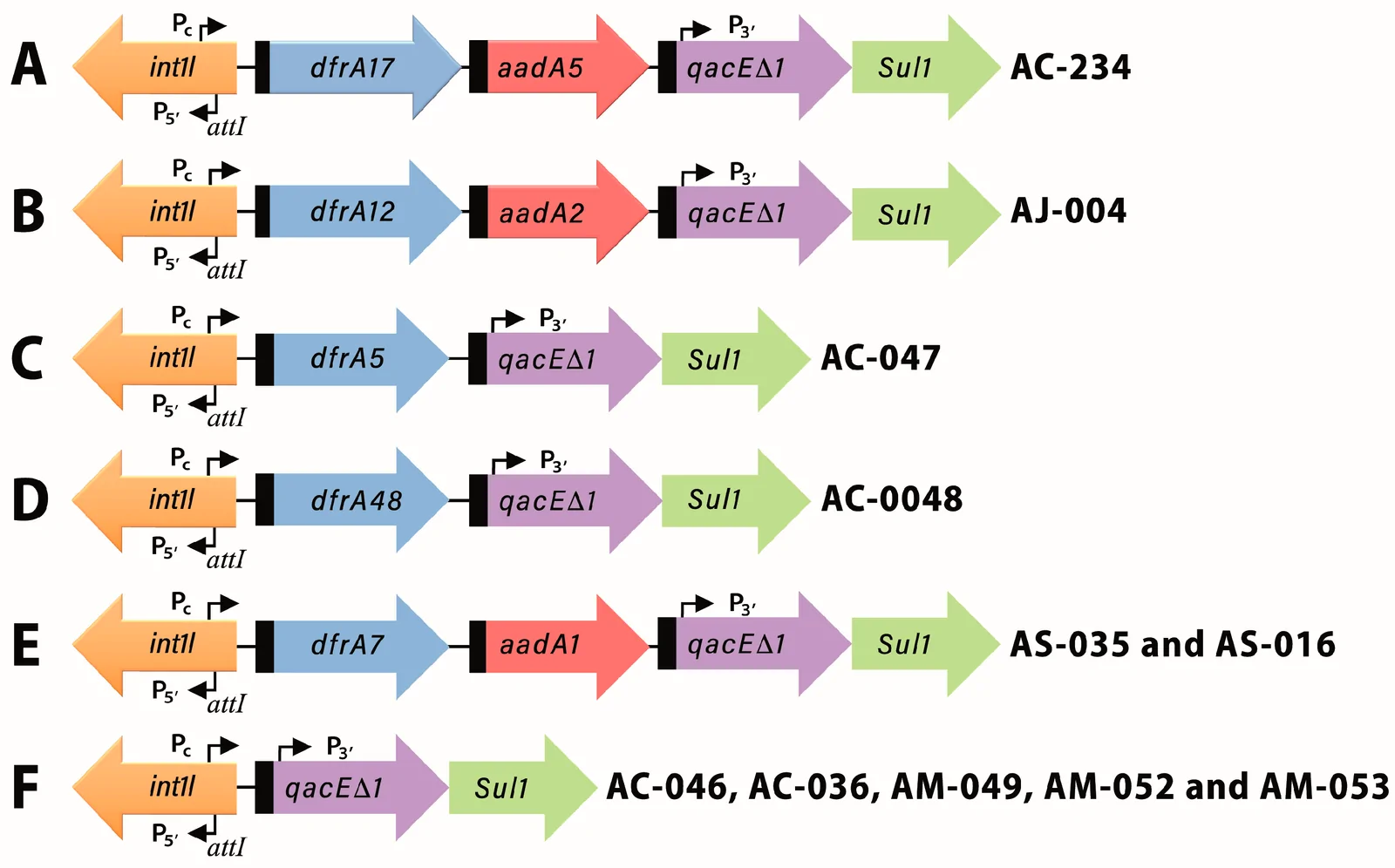
Schematic representation of the genetic organization of class 1 integrons detected in *Aeromonas* spp. isolates recovered from HWW. Six class 1 integron configurations (**A**–**F**) were identified, comprising five cassette-containing arrays and one cassette-free configuration. The corresponding isolate codes are indicated for each integron structure. Gene cassettes *dfrA* encode trimethoprim resistance, whereas *aadA* cassettes encode aminoglycoside resistance.

**Figure 3:**
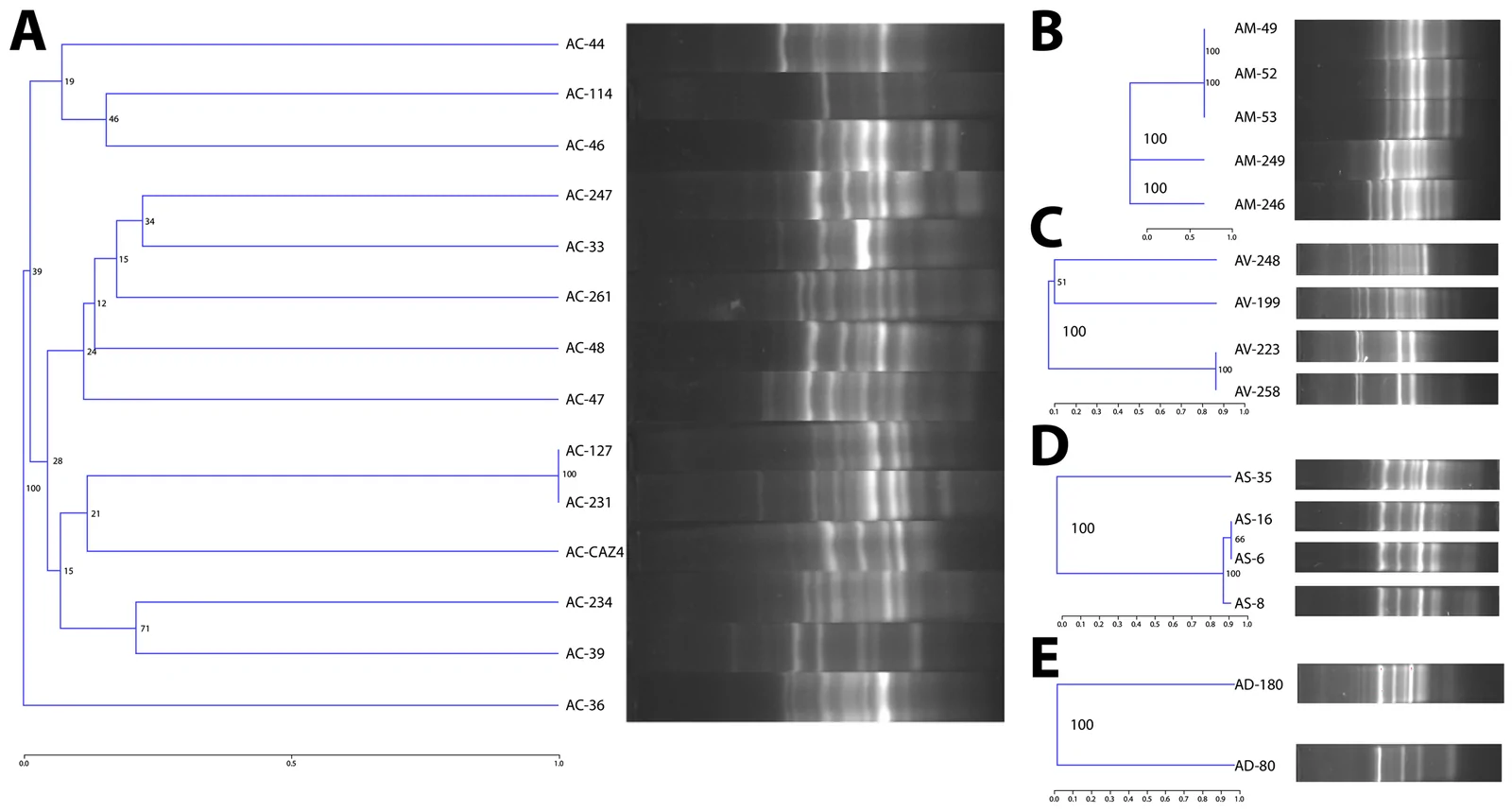
ERIC-PCR fingerprinting and dendrogram analysis of *Aeromonas* spp. isolates. Dendrograms were generated using the Dice similarity coefficient and the UPGMA clustering method. Panels correspond to (**A**) *A. caviae*; (**B**) *A. media*; (**C**) *A. veronii*; (**D**) *A. salmonicida*; (**E**) and *A. dhakensis*. Representative ERIC-PCR banding patterns are shown alongside each dendrogram.

**Table 1:** Primers used in this study.

Primer	Molecular Target	Sequence (5′→3′)	Size (bp)	Reference
AERO-F	*rpoD*	YGARATCGAYATCGCCAARCGB	770–782	[32]
AERO-R		GRCCDATGCTCATRCG RCGGTT		
KPC-F	*bla_KPC_* family	CATTCAAGGGCTTTCTTGCTGC	538	[33]
KPC-R		ACGACGGCATAGTCATTTGC		
OXA-48F	*bla_OXA-_*_48_ like	GCTTGATCGCCCTCGATT	281	
OXA-48R		GATTTGCTCCGTGGCCGAAA		
VIM-F	*bla_VIM_*	GATGGTGTTTGGTCGCATA	390	
VIM-R		CGAATGCGCAGCACCAG		
IMP-F	*bla_IMP_*	TTGACACTCCATTTACDG	139	
IMP-R		GATYGAGAATTAAGCCACYCT		
SHV-F	*bla_SHV_*	AGCCGCTTGAGCAAATTAAAC	713	
SHV-R		ATCCCGCAGATAAATCACCAC		
CTX-M-G1-F	*bla_CTX-M_* group 1	TTAGGAARTGTGCCGCTGYA	688	
CTX-M-G1-R		CGATATCGTTGGTGGTRCCAT		
NDM-F	*bla_NDM_*	GGTTTGGCGAT CTGGTTTTC	621	[34]
NDM-R		CGGAATGGCTCATCACGATC		
mcr1_320bp_fw	*mcr-1*	AGTCCGTTTGTTCTTGTGGC	320	[35]
mcr1_320bp_rev		AGATCCTTGGTCTCGGCTTG		
IntL-F	Integrase *intll*	GTTCGGTCAAGGTTCTG	923	[36]
IntL-R		GCCAACTTTCAGCACATG		
qacEΔ1-F	*qacEΔ1-sul1*	ATCGCAATAGTTGGCGAAGT	800	
sul1-B		GCAAGGCGGAAACCCGCGCC		
In-F	Variable region	GGCATCCAAGCAGCAAGC	Variable	
In-B		AAGCAGACTTGACCTGAT		
ERIC1R	Intergenic consensus	ATGTAAGCTCCTGGGGATTCA	Variable	[37]
ERIC2		AGTAAGTGACTGGGGTGAGC		

## Data Availability

Bello-López, Juan Manuel (2026), “Surveillance of hospital wastewater reveals MDR and XDR *Aeromonas* spp. harboring clinically relevant β-lactam resistance genes and class 1 integrons”, Mendeley Data, V1, https://doi.org/10.17632/ctz22xh7v9.1.
